# Quality of leadership and self-rated health: the moderating role of ‘Effort–Reward Imbalance’: a longitudinal perspective

**DOI:** 10.1007/s00420-022-01941-w

**Published:** 2022-12-07

**Authors:** Marco Kuchenbaur, Richard Peter

**Affiliations:** Department of Medical Sociology, Institute of the History, Philosophy and Ethics of Medicine, University of Ulm, Ulm, Germany

**Keywords:** Quality of Leadership, Self-rated health, Effort–Reward Imbalance, Moderation, Linear mixed model of change, Longitudinal study

## Abstract

**Objective:**

Longitudinal studies on the influence of leadership behavior on employees’ self-rated health are scarce. As a result, potential mechanisms describing the impact of leadership behavior on health have not been adequately investigated so far. The present study accounts for the influence of leadership behavior on self-rated health within the framework of the Effort–Reward Imbalance model.

**Methods:**

The study was conducted on the basis of a cohort which comprised a random sample of healthcare workers from ten different hospitals and one elderly nursing home in Germany. A 2-level repeated measurement model with random intercept and slopes was modeled, since it was aimed to account for individual as well as intra-individual variation of subjective health across three time points over 36 months. Beside ‘Effort–Reward Imbalance’ and ‘Quality of Leadership’ from the Copenhagen Psychosocial Questionnaire, physical and mental health was assessed by German version of the SF12 multipurpose short-form measure of health status.

**Results:**

‘Effort–Reward Imbalance’ and a lack in ‘Quality of Leadership’ negatively affect self-rated physical health. No effect was found for self-rated mental health. Effort–Reward Imbalance significantly moderates the effect of ‘Quality of Leadership’ on self-rated physical health.

**Conclusion:**

The findings, and the interaction effects in particular, suggest that leadership behavior moderated by factors such as appreciation and support, influences self-rated physical health. The study therefore provides an interpretation for leadership behavior and its influence on employees’ self-rated health within the ‘Effort–Reward Imbalance’ model.

**Supplementary Information:**

The online version contains supplementary material available at 10.1007/s00420-022-01941-w.

## Background

Recent meta-analyses have shown that psychosocial hazards at worksite have an impact on both the physical, e.g., coronary heart diseases (Taouk et al. 2020) and mental health, e.g., psychological distress and depression (van der Molen et al. 2020) of employees.

Leadership in its function to influence other people (Haslam et al. 2015) plays an important role in the framework of psychosocial hazards at worksite. It is considered as relevant for the wellbeing and health of employees (Montano et al. 2017; Skakon et al. 2010; Harms et al. 2017; Cummings et al. 2018; Kuoppala et al. 2008). Due to heterogeneity in existing literature and a variety of conceptualizations of leadership are associated with health (Nyberg et al. 2005), leadership behavior has only been defined as the quality of the next higher managers’ leadership in different contexts and domains (Burr et al. 2019). By taking this generic approach, the intention is to account for as many facets of leadership behavior as possible.

There is a large body of literature on the role of leadership behavior and style as a psychosocial risk factor for employees’ health and well-being, but longitudinal studies in particular are scarce (Montano et al. 2017). Moreover, the conceptualizations of leadership as a construct are also very heterogeneous. Research has focused on particular traits of leaders as well as behaviors and styles (Nyberg et al. 2005). Characteristics of leaders manifests and affects a variety of levels of social interaction (Montano 2016). As a consequence, depending on the conception, leadership characteristics have protective but also risk-amplifying effects on the development of physical and mental health. So-called destructive leadership behaviors (Schyns and Hansbrough 2010), which manifest in abusive and manipulative behaviors of leaders, are linked to lower mental health and well-being (Schyns and Schilling 2013). On the other hand, there are leadership styles, e.g., ‘transformational leadership style’ that seem to have a protective effect on the mental health and well-being of employees (Nielsen et al. 2008). In terms of physical health, this protective effect is reported in relation to ischemic heart disease (Nyberg et al. 2009). Positive leadership behavior has also been reported to have protective effects on mental health (Madsen et al. 2014). What mechanisms may be associated with these findings?

Social support as a stress buffer can operate as active coping assistance through encouragement as well as through information and advice (Madsen et al. 2014). By providing employees with a sense of mattering, self-esteem, and belonging (Thoits 2011), supervisors may influence physiological arousal and distress by their function as similar others within interactions. Polite and considerate treatment by supervisors may be functional for the experience of control and support. In situations in which individuals have no direct control, for example within hierarchical structures in the workplace, positive self-experience can be made in the form of opportunities to exert influence, appreciation and support. In many aspects of life, individuals do not have direct control over mechanisms of change and therefore have to rely on proxy control to change their lives for the better. A leadership behavior which accounts for considerate and polite treatment in the context of the workplace can be regarded as a form of proxy control (Bandura 2012). Supervisors impact a psychosocial environment in which their employees can have positive self-experience and which consequently may influence their health and well-being.

Positive self-experiences triggered by positively connoted reciprocal relationships are contingent on a psychosocial environment in which experiences of belonging, acting, contributing and giving feedback, can be made by employees. The model of ‘Effort–Reward Imbalance’ (ERI) (Siegrist 1996a) describes these factors and their relationships in detail. Within the model of ERI, psychosocial environments are characterized by interpersonal relationships, based on a norm mutual cooperative investments, i.e., efforts and the expectancy of a response to these efforts, i.e. rewards. If this norm of reciprocity is violated on a frequent basis, the imbalance of effort and reward leads to a state of emotional distress and a negative self-experience (e.g., low self-esteem). In contrast, a psychosocial environment characterized by appreciation and support promotes positive self-experience and the feeling of control and successful agency which can be conducive to health and well-being (Siegrist and Marmot 2004). The absence of an experience of control and support can lead to adverse health effects: an imbalance of mutual commitment between employer and employee can influence strong negative emotions. This experience tends to sustained autonomic and neuroendocrine activation which links experiences of imbalanced social reciprocity to development of physical and mental diseases (Siegrist 2005), for example coronary heart disease (Dragano et al. 2017).

Studies that have previously examined the relationship between the mental and physical health of employees and the behavior of their supervisors suffer from a certain number of limitations. Beside a lack in longitudinal perspectives mentioned above, confounding variables like age, gender and workplace contexts, as well as an investigation of underlying mechanisms of the relationship between leadership and health were not considered (Montano et al. 2017; Harms et al. 2017). Some studies only surveyed the general state of health and not its physical and mental health components (Schmidt et al. 2018). Accordingly, a more established and detailed health status instrument was used for this study. The SF12 multipurpose short-form measure of health status offers a way to measure eight commonly represented concepts of health (Nübling et al. 2006) By locating leadership within the etiologically sound framework of the model of ‘Effort–Reward Imbalance’, an interpretation of the impact of leadership on physical and mental health will be provided. This study in particular investigates changes in both components of general health (physical and mental) over three time-points by focusing on the perceptions of quality of leadership in a cohort of healthcare workers. This study therefore rather focusses on specific manifestations of leadership behavior relevant to physical and psychological subjective health. Our first hypothesis is:

H1: Effort–Reward Imbalance (a) and lack in Quality of leadership (b) have a negative impact on self-rated physical and mental health over time.

Based on the mechanisms of control and support described above, we hypothesize that leadership quality interacts with ERI. As leadership quality is strongly associated with dimensions of social support and recognition by supervisors (ibid.) it is expected to moderate the impact of ERI on health. Thus, taking into account the mechanisms of ERI, an explanation has yet to be offered as to why leadership behavior influences subjective health (Montano et al. 2017):

H2: Effort–Reward Imbalance amplifies the experience of lack in quality of leadership and therefore the impact on self-rated physical and mental health.

The aim of this study is to investigate the influence of leadership quality on subjective physical and mental health. An interaction effect with the ERI model is suspected.

## Methods

To test these assumptions, a cohort of healthcare workers was used. The cohort was surveyed at three time points over a period of 36 months to assess psychosocial hazards at worksite as well as subjective physical and mental health. All effects are modeled by using two-level ‘Linear Mixed Effect Models’ (Hoffman 2015) under the assumption of conditional growth.

### Study design and sample

The study sample was taken from the HALT geben study, which aims to reduce healthcare worker’s physical and psychosocial workload (Montano et al. 2020). The study was designed as cluster-randomized intervention study. It surveyed participants in the cohort with a questionnaire at three time points with an interval of 12 months. The cohort comprised a random sample of healthcare workers from ten different hospitals and one elderly nursing home. Between healthcare workers in the hospitals and those in the elderly nursing home no significant differences in the perception of psychosocial hazards have been found. Eligible participants in the survey, were required to be health care workers, older than 18 years of age, and predominantly work in a single ward. All employees of the eleven facilities were contacted via mail. Participants were asked to give written consent before enrolling them. According to variation in cluster sizes, a sampling schedule proportional to the cluster sizes was established. The allocation was carried out by simple random sampling (Montano et al. 2020). In total, 450 participants who agreed to participate, received a questionnaire at baseline t1 after randomization.

**Fig. 1:**
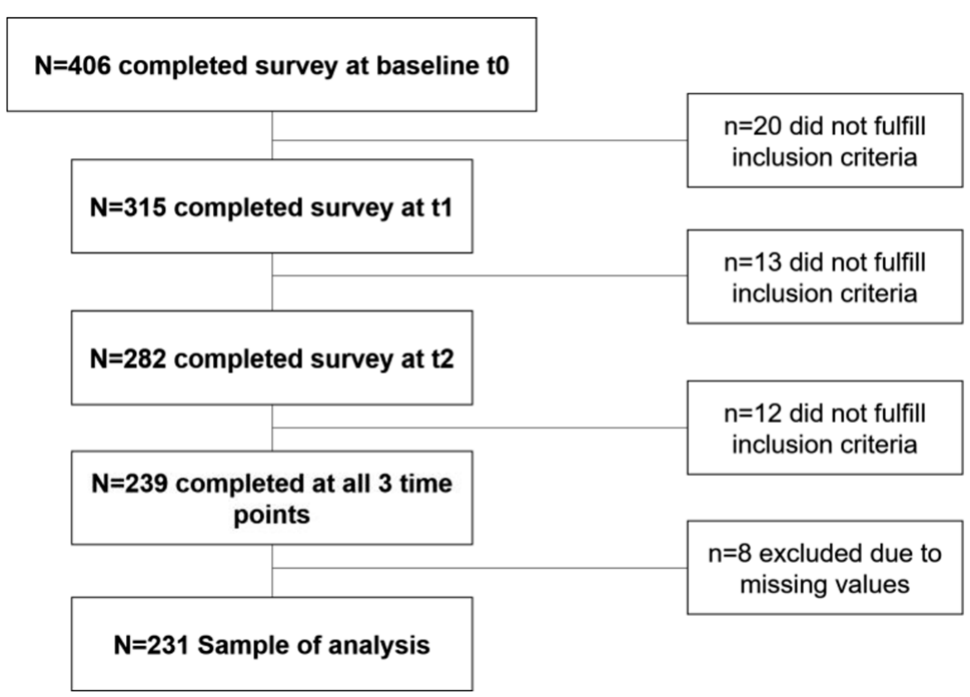
Flowchart of the ‘HALTgeben’ cohort (*N* = 231)

The sample of analysis consisted only of all cohort participants who responded to the survey at all three time points ($N=231$) (see Fig. 1). Of these individuals, $19.2 \%$ were under age 40, $52.7 \%$ were under age 55, and $28 \%$ were over age 55 at third time point.84.9 $\%$ of the participants were female (see Table 1).

**Table 1:** Cohort characteristics: statistical comparisons over three time points (*N* = 231)

Min/max		*t*0		*t*1		*t*2		Test^2^
		Mean/percent	SD	Mean/percent	SD	Mean/percent	SD	
Age								${\chi }^{2}$ = 0.876
Up to under 40		20.40%		18.50%		20.80%		
Up to under 55		53.90%		52.80%		53.70%		
Over 55		25.70%		28.70%		25.50%		
Gender								${\chi }^{2}$ = 0.042
Male		15%		14.80%		14.40%		
Female		85%		85.20%		85.60%		
Education								${\chi }^{2}$ = 0.087
Higher education (12 years)		27.20%		25.90%		26.40%		
Lower education (under 12 years)		72.80%		74.10%		73.60%		
Physical health (SF12)	14.1/63.4	43.92	8.865	42.806	8.949	42.065	9.198	*F* = 2.26
Mental health (SF12)	20.8/70.3	45.767	10.796	45.361	9.814	45.104	10.45	*F* = 0.219
Quality of leadership^1^	1/5	3.209	0.914	3.132	0.946	3.089	0.948	*F* = 0.883
Effort–Reward Imbalance	0.3/3.4	0.905	0.35	0.857	0.383	0.844	0.372	*F* = 1.608
ERI Overcommitment	¼	2.6	0.608	2.622	0.587	2.632	0.622	*F* = 0.147
Work-privacy conflict	1/4	2.704	0.862	2.604	0.842	2.65	0.873	*F* = 0.711
Worksite characteristics								${\chi }^{2}$=0.121
Intensive care/surgery		38.80%		40.30%		40.30%		
Other		61.20%		59.70%		59.70%		

*SD* standard deviation

Statistical significance: *p < 0.1; **p < 0.05; ***p < 0.01

^1^Negatively poled: higher values indicate lower quality

^2^Statistical Tests: ${\chi }^{2}$-Test and ANOVA (analysis of variance)

### Measures

Sociodemographic information was collected at all three time points (age, gender), and in some cases only at the first time point (education). Because scales of COPSOQ and ERI cover a wide range of dimensions of psychosocial hazards at worksite (Formazin et al. 2014), they were used in combination. Information on all COPSOQ scales used, can be found in the supplementary file of the study protocol (Montano et al. 2020). A correlation matrix of the scales used in this study can be found as online supplementary information (Supplementary Table S1).

### Effort–Reward Imbalance

All three German version subscales were used to assess ‘Effort–Reward Imbalance’ (Siegrist et al. 2004; Siegrist 1996b) (‘Effort’: $\alpha =0.80$, original: $\alpha =0.79$; ‘Reward’: $\alpha =0.79$, original: $\alpha =0.85$; ‘Overcommitment’:$\alpha =0.57$, orginal: $\alpha =0.79$). Subscales were measured at all three time points. All items were assessed on a 4-point Likert-scale ranging from 1 = ”not at all” to 4 = ”very strong”. For better interpretability in the statistical analysis, the subscale ‘Reward’ was negatively poled afterwards.

### Copenhagen psychosocial questionnaire

The German Version of COPSOQ (Nübling et al. 2005) was used to assess information at all three time points. ‘Quality of Leadership’ ($\alpha =0.92$, original: $\alpha =0.89$) was assessed on a five-point Likert-scale ranging from 1 = “to a very high extent” to 5 = ”to a very low extent”. For better interpretability in the statistical analysis, the scale was negatively poled so that higher values represent lower leadership quality and vice versa. ‘Work-privacy conflict’ ($\alpha =0.83$, original: $\alpha =0.90$) was assessed on a five-point Likert-scale ranging from 1 = “to a very high extent” to 5 = ”to a very low extent”.

### Physical and mental health (SF12)

Physical and mental health were assessed by using the German version of the SF12 multipurpose short-form measure of health status (Nübling et al. 2006; Ware et al. 1995) which is also used for the ‘Socio-Economic Panel’ (SOEP). The two subscales ‘Physical component summary’ ($\alpha =0.82$, original:$\alpha =0.89$) and ‘Mental component summary’ ($\alpha =0.80$, original:$\alpha =0.76$) were assessed on a five-point Likert-scale ranging from 1 = “always” to 5 = ”never” or ranging from 1 = ”strong” to 3 = ”not at all”, respectively. Four single items were transformed directly to the range of 0–100, for subscales with two items a mean value of the both was calculated (arithmetic mean) (ibid.).

### Statistical analyses

Two-level linear mixed effect models (LMM) with repeated measurement were estimated according to the longitudinal study design and continuous outcome variables. In comparison to repeated measurement ANOVA (Analysis of variance), one advantage is the estimation of effects with missing measurement points. Moreover, individually varying trajectories can be estimated for each subject (West 2014). A restricted maximum likelihood (REML) method was used to estimate the variance components because this method provides more accurate estimates than Maximum likelihood (MLE) estimation (Chen and Chen 2021; Hoffman 2015). All model estimates were adjusted for the confounding effects of age, gender, education, and workplace characteristic. For the analysis, a general structure for the random effect variance–covariance matrix, that allows the random intercepts and slopes to have different variances and to be correlated (Gałecki and Burzykowski 2013), is assumed. As this study is interested in observing a trend rather than a contrast, we formulated the following LMM as a multilevel model, where level 1 predicts variation within subjects over time and level 2 predicts variation between subjects. The specification of the null model will be as follows:

Level 1:

$${y}_{ti}={\beta }_{0i}+{\beta }_{1i}\left({\mathrm{Time}}_{ti}\right)+{e}_{ti}$$

Level 2:

$${\beta }_{0i}={\gamma }_{00}+{U}_{0i}$$

$${\beta }_{1i}={\gamma }_{10}+{U}_{1i}$$

Composite:

$$y_{ti} = \left( {\gamma_{00} + U_{0i} } \right) + \left( {\gamma_{10} + U_{1i} } \right)\left( {{\text{Time}}_{ti} } \right) + e_{ti} .$$

Interaction effects were statistically examined by applying ‘Simple slope analysis’ (Aiken and West 2010) and graphically by generating ‘Johnson-Neyman plots’ (Bauer and Curran 2005). The scales of the psychometric instruments were calculated only if more than 70% of the items defining the scale were answered by the respondent. This assumption states that the missing 30% of the items are missing at random (MAR). The proportions of missing items are rounded up to the nearest integer (Schafer and Graham 2002). Calculations were performed with the statistical environment R, using the package “lme4” (Bates et al. 2015) to perform LMM analyses. Models’ performances were evaluated with the package “performance” (Lüdecke et al. 2021).

## Results

### Descriptive results

For physical health, a comparison of means (ANOVA) showed a significant change over the three time points of measurement. The result showed that modeling a trend in change of physical health over time could be promising. Other characteristics remained stable over time. The mental health of the participants did not change significantly over time, which is why the modeling did not yield any results.

### Main effects

While checking the unconditional models (null model), time was found to be at least a fixed effect. The likelihood ratio test, comparing the null models showed no difference between the null model for a fixed or a fixed and random time effect, however, between both and the empty means random intercept model there was found a significant difference (${\chi }^{2}=15.44 [p<.001]$). Nevertheless, a consideration of time as random effect seems plausible, since self-reported health can change differently over time. The aim of the hypothesized model is to reflect both the average and the individual change in self-reported health over time. In the null model with fixed and random time effect, the $ICC=0.68$ indicates that $32\%$ of the variation lies within individual variance over time.

A conditional growth LMM (estimated using REML) was fitted to predict physical and mental health. The model included time and subject as random effects. Since modeling mental health in the context of ERI and leadership behavior has not shown results, only modeling physical health is considered below.

The total explanatory power of the model explaining physical health is substantial (conditional ${R}^{2}=0.67$) and the part related to the fixed effects alone (marginal ${R}^{2}$) is of 0$.14$. The model’s intercept is at $67.36 (95\% CI \left[60.25, 74.48\right], p<0.001$). For the main effects of interest, ‘Effort–Reward Imbalance’ $-7.56 (95\% CI \left[-14, -1.11\right], p=0.022)$ and lack in ‘Quality of Leadership’ $-2.11 (95\% CI \left[-3.64, -0.59\right], p=0.007)$ a negative significant effect can be reported. The interaction term of both, ERI and ‘Quality of Leadership’ is also significant and positive $1.55 (95\% CI \left[0.01, 3.9\right], p=0.049)$ (see Table 2). Model 2 was adjusted for several effects, with age $-1.17 (95\% CI \left[-1.66, -0.67\right], p<.001)$, education $-2.18 (95\% CI \left[-4.31, -0.05\right], p=0.045)$, and work-family conflict $-1.67 (95\% CI \left[-2.49, -0.84\right], p<0.001)$ which significantly contributing in explaining the decline in physical health over time. To test which model fits the data better, a Likelihood ratio test was performed which showed that the adjusted model (Model 2) fits the data significantly better (${\chi }^{2}=42.08 [p<0.001]$) than the unadjusted model (Model 1). Due to this result, Model 2 was adopted.

**Table 2:** Linear mixed effect models for dependent variable physical health (*N* = 231)

Fixed effects	Model 0			Model 1			Model 2^2^		
	Estimates	CI	*p*	Estimates	CI	*p*	Estimates	CI	*p*
(Intercept)	43.74	42.61–44.88	** < 0.001**	55.03	49.27–60.79	** < 0.001**	67.36	60.25 – 74.48	** < 0.001**
Time	− 0.92	− 1.46 to − 0.39	**0.001**	− 1.11	− 1.65 to − 0.56	** < 0.001**	− 1.13	− 1.66 to − 0.59	** < 0.001**
Quality of leadership^1^				− 2.56	− 4.10 to − 1.02	**0.001**	− 2.11	− 3.64 to − 0.59	**0.007**
Effort–Reward Imbalance				− 9.74	− 16.00 to − 3.48	**0.002**	− 7.56	− 14.00 to − 1.11	**0.022**
Quality of leadership x Effort–Reward Imbalance				1.89	0.34–3.44	**0.017**	1.55	0.01–3.09	**0.049**
Random effects									
σ^2^	27.84			27.22			27.44		
τ_00_	50.48 _id_			46.36 _id_			40.68 _id_		
τ_11_	1.34 _id.zeit_			1.66 _id.zeit_			0.96 _id.zeit_		
ρ_01_	0.08 _id_			0.08 _id_			0.03 _id_		
ICC	0.66			0.65			0.61		
N	231 _id_			231 _id_			231 _id_		
Observations	638			638			638		
Marginal R^2^/conditional R^2^	0.007/0.662			0.044/0.666			0.144/0.665		

CI confidence interval at the 95% level

Statistical significance: *p < 0.1; **p < 0.05; ***p < 0.01

^1^Negatively poled: higher values indicate lower quality

^2^Adjusted for age, gender, worksite characteristic, education, overcomittment, work-family conflict

### Moderation effects

A test on ‘Simple slopes’ (Aiken and West 2010) at specific levels of the predictors (− 1 SD, mean, + 1 SD) was performed to examine moderation effects of lack of ‘Quality of Leadership’ on the relationship of ERI on physical health. This approach tests for the effect of the moderator variable at different, designated levels on the outcome variable while holding the predictor variable constant (Bauer and Curran 2005).

**Fig. 2:**
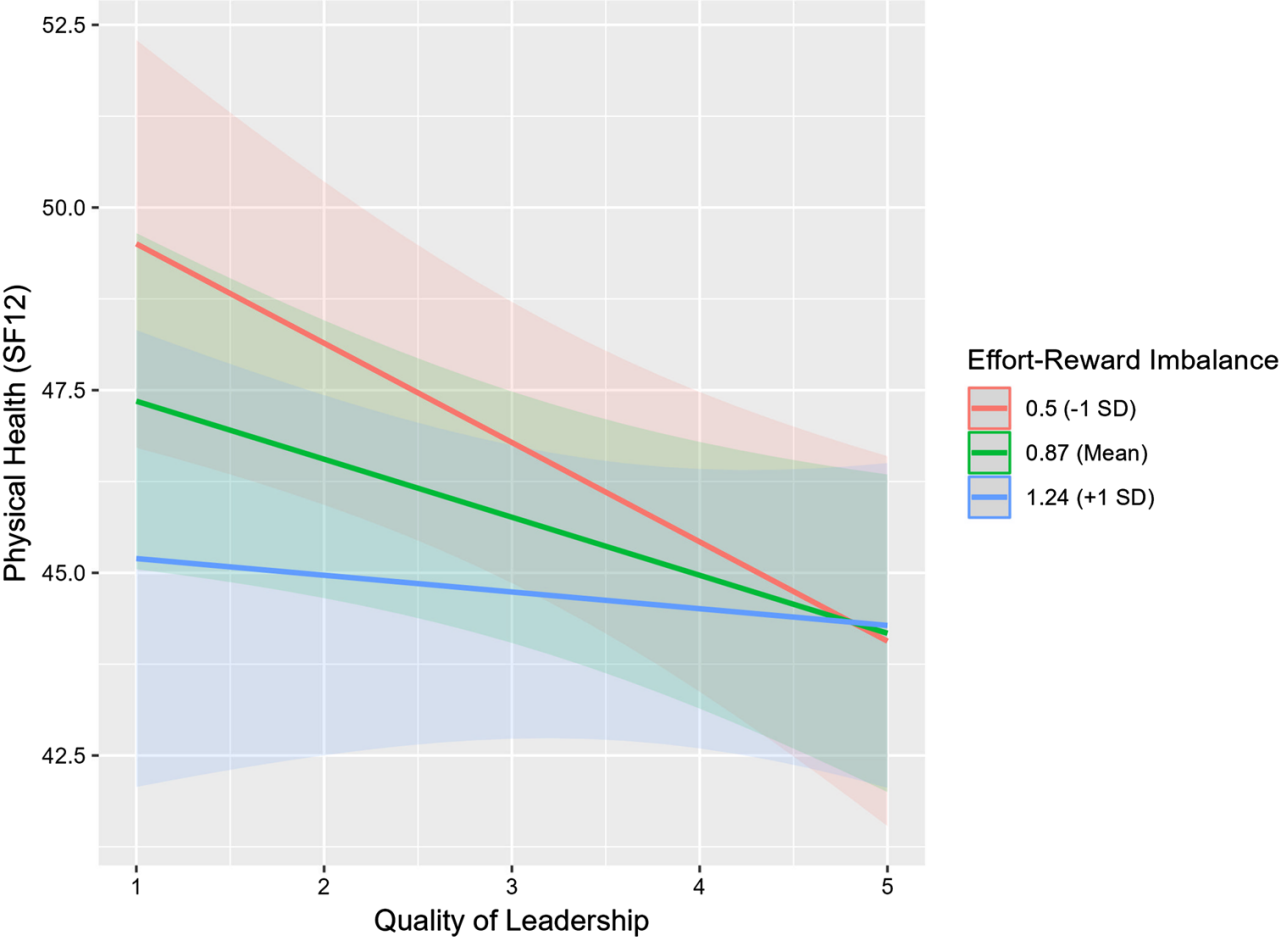
Moderation effect of Model 2 (*N* = 231). Moderation effect of ERI and ‘Quality of Leadership’ on physical health: the worse the ‘Quality of Leadership’, the less negative the effect of ERI on physical health. ‘Quality of Leadership’: higher values indicate lower quality.*SD* standard deviation

The negative impact of ERI on physical health was significantly stronger in cases where employees reported a better quality of leadership (− 1 SD below average): $-4.21 ( p=0.017)$ (see Table 3, Fig. 2). Consequently, the worse the ‘Quality of Leadership’ behavior becomes, the less ERI impacts physical health negatively. Or in other words, if subjects are already experiencing an ‘Effort–Reward Imbalance’, leadership quality is no longer significantly affecting the relationship of ERI and physical health.

**Table 3:** Moderation analysis via ‘Simple slope analysis’ of Model 2 (*N* = 231)

Level	Effort–Reward Imbalance	Quality of leadership^1^	Physical health	SE	*df*	*t* value	*p*		Sig.
−1 SD	0.49	Fixed	− 1.38	0.474	599.286	− 2.908	0.003	^**^	
Mean	0.86	Fixed	− 0.79	0.367	600.346	− 2.169	0.030	^*^	
+ 1 SD	1.23	Fixed	− 0.21	0.465	588.033	− 0.461	0.645		
−1 SD	Fixed	2.20	− 4.21	1.758	609.300	− 2.396	0.017	^*^	
Mean	Fixed	3.14	− 2.73	1.255	614.349	− 2.180	0.029	^*^	
+ 1 SD	Fixed	4.07	− 1.26	1.076	600.518	− 1.170	0.242		

*SE* standard error, *df* degrees of freedom, *Sig* significance

Statistical significance: *p < 0.1; **p < 0.05; ***p < 0.01

^1^Negatively poled: higher values indicate lower quality

## Discussion

The current study used a LMM to model a linear trend in physical and mental health as a function of ERI and ‘Quality of Leadership’. Results from the multilevel model have shown that ERI and lack in ‘Quality of Leadership’ have an adverse effect on physical health. For mental health these assumptions were not statistically significant. Furthermore, lack in ‘Quality of Leadership’ moderates the effect of ERI on physical health. The moderating relationship between ERI and lack in ‘Quality of Leadership’ offers an interpretation for the effect of leadership behavior on self-reported physical health. The results are in line with findings of previous studies, but go beyond in particular. Leadership behavior that’s not supportive, appreciative and well organized has a negative impact on physical health. (Montano et al. 2017; Harms et al. 2017; Skakon et al. 2010). Vice versa, another study that is focusing on a cross-professional perspective show that certain forms of leadership behavior can reduce the perception of an ‘Effort–Reward Imbalance’ in employees (Weiß and Süß 2016).

In comparison to previous studies, the current study uses validated instruments for the assessment of self-rated health and applies a longitudinal study design to answer the question on how leadership behavior affects subordinates’ health (Schmidt et al. 2018).

### Possible mechanisms

#### Relationship of leadership quality and physical health

Results from the moderation analysis showed that participants’ perception of an increasingly worsening leadership quality has negatively influenced the self-rated physical health as long as no ‘Effort–Reward Imbalance’ exists. According to Formazin et al., this suggests that there are certain dimensions of interpersonal relations in ‘Quality of Leadership’ which in turn cover dimensions of the ‘Effort–Reward Imbalance’ model (Formazin et al. 2014). The sub dimension of ‘Rewards’ in the ERI model is primarily characterized by factors of support and appreciation. As mentioned above, these forms of rewarding leadership behavior may foster a sense of mattering, belonging, and self-esteem (Thoits 2011), which positively affects neuronal and endocrine activation patterns. This in turn may have influenced perceptions of self-rated physical health to the positive or negative (Siegrist 2005).

### Moderation effect

Notably, among individuals with ‘Effort–Reward Imbalance’ physical health was significantly worse in cases where better leadership quality was reported than among individuals experiencing a lower ‘Quality of Leadership’ without having an ‘Effort–Reward Imbalance’. Thus, physical health worsened more in cases where a better ‘Quality of Leadership’ was reported while Effort–Reward Imbalance was experienced. An increasingly lower ‘Quality of Leadership’ no longer plays a significant role in explaining a worsening in physical health over time, once individuals experience an imbalance of efforts and rewards.

One possible explanation for this relationship is that certain stress constellations between effort and reward may be so strong that ‘Quality of Leadership’ as a possible buffering resource (Cohen and Wills 1985) cannot influence the negative relationship between ERI and physical health. Results similar to these were reported by Schmidt et al. where they tested the moderation effect of job strain on the association between supportive leadership behavior and self-reported health (Schmidt et al. 2018). Harms et al. found that employees who are highly stressed are less likely to report a strong exchange between themselves and their supervisors (Harms et al. 2017). Vice versa, when ‘Quality of Leadership’ is very low, other stressors from the ‘Effort–Reward Imbalance’ framework become less relevant for explaining self-rated health because they are absorbed by this effect. This is an indicator of the potential, overall relevance of leadership behavior for employees’ self-rated health.

### Relevance

The findings are relevant to better understand the mechanisms by which leadership behavior affects self-rated health. The framework of the ‘Effort–Reward Imbalance’ model offers an interpretation for this mechanism, which has not yet been used in any previous study. The interaction effects contribute to a better understanding of the relationship between different factors of the psychosocial environment at worksite. Potentially, this may also provide guidance for interventions to improve self-rated health among employees. Based on our findings, an intensive training for supervisors on how to interact with their employees is recommended as a first step to improve employee’s health.

### Strength and limitations

There are some limitations that should be discussed in the context of this study. No control for biomedical factors (Body-Mass-Index, comorbidity) or behavioral factors (sport, alcohol consume. smoking etc.) was conducted. Instead, a number of psychosocial factors were taken into account which were not considered in other studies. Due to repeated measurement, the problem of overestimation of effects of self-reported health and psychosocial risk factors is rather small. Another possible limitation is the restriction of the cohort to health care professionals, in this case nurses. A comparison with other branches has shown that nurses report a stronger imbalance of effort and reward than other professions (Bakker et al. 2000). In addition, the use of a generic instrument as the COPSOQ Questionnaire can be seen critically, as the ‘Quality of Leadership’ subscale does not cover all specific aspects of leadership behavior. On the other hand, this can be seen as a strength, as it is an attempt to offer a more general framework of assessment of leadership behavior that is not limited to specific research traditions and definitions of leadership. Due to low Cronbach’s alpha for the subscale ‘Overcommitment’ an interpretation of this variable is only possible to a limited extent.

However, the present results do offer an interpretation of the relationship between leadership behavior and self-rated health. The moderation effect with ‘Effort–Reward Imbalance’ identified in the study has not been described before. Additionally, the models in this study were adjusted for various confounding variables. Age, gender and education were taken into account, along with worksite characteristics (i.e. type of ward) (Montano et al. 2017).

## Conclusion

It has been shown that ‘Quality of Leadership’ as a part of the psychosocial environment at workplace addresses, among other aspects, factors of appreciation and support. This is suggested by the interaction between ERI and ‘Quality of leadership’. As long as no imbalance between efforts and rewards is perceived, the quality of leadership negatively influences self-reported physical health. Moderation analysis showed that, once an imbalance of ‘Efforts’ and ‘Rewards’ is perceived, the lack of ‘Quality of Leadership’ no longer has a statistically significant influence on self-rated physical health. A potential approach in the light of these findings is, for example, to provide extensive training for supervisors on how to interact with their employees.

## Supplementary Information

Below is the link to the electronic supplementary material.

Supplementary file1 (JPEG 357 kb)

Supplementary file2 (DOCX 17 kb)

## Data Availability

The datasets generated during and/or analyzed during the current study are available from the corresponding author on reasonable request.

## Code availability

The code used for analysis of the dataset is available from the corresponding author upon request.
