# Supplementary figures and images for: Quality of leadership and self-rated health: the moderating role of ‘Effort–Reward Imbalance’: a longitudinal perspective
Source: Int Arch Occup Environ Health. 2022 Dec 7;96(3):473–82. doi: 10.1007/s00420-022-01941-w (PMC9968269; doi:10.1007/s00420-022-01941-w)

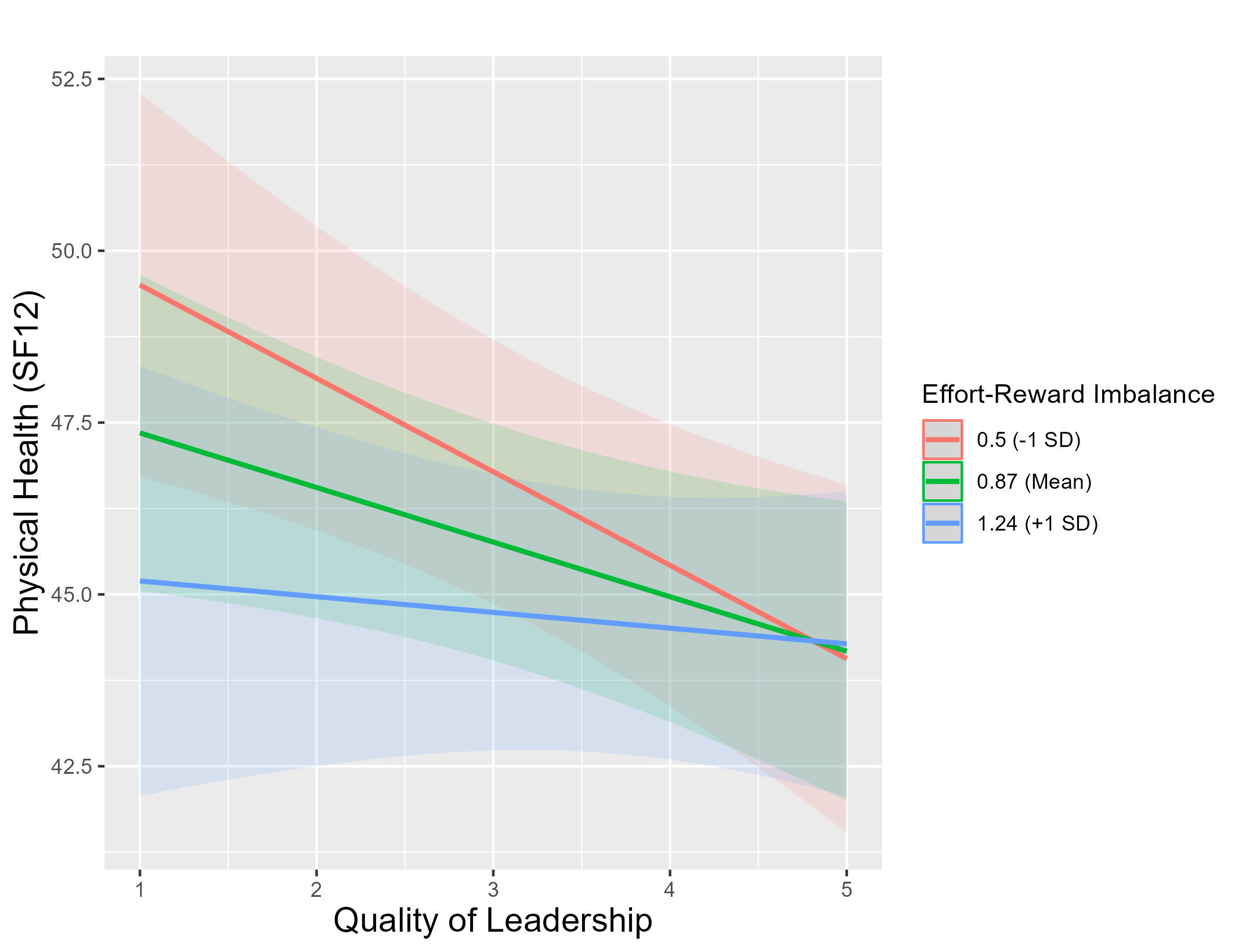

Supplement: Supplementary file 1 — Supplementary file1 (JPEG 357 kb) [file 420_2022_1941_MOESM1_ESM.jpeg]
