# Supplementary material for: Quality of leadership and self-rated health: the moderating role of ‘Effort–Reward Imbalance’: a longitudinal perspective
Source: Int Arch Occup Environ Health. 2022 Dec 7;96(3):473–82. doi: 10.1007/s00420-022-01941-w (PMC9968269; doi:10.1007/s00420-022-01941-w)
Supplement: Supplementary file 2 — Supplementary file2 (DOCX 17 kb) [file 420_2022_1941_MOESM2_ESM.docx]

| Variable | *M* | *SD* | 1 | 2 | 3 | 4 | 5 |
| --- | --- | --- | --- | --- | --- | --- | --- |
|  |  |  |  |  |  |  |  |
| 1. Effort-Reward-Imbalance | 0.87 | 0.37 |  |  |  |  |  |
|  |  |  |  |  |  |  |  |
| 2. Quality of Leadership^1^ | 3.13 | 0.94 | .36** |  |  |  |  |
|  |  |  | CI [.29, .42] |  |  |  |  |
|  |  |  |  |  |  |  |  |
| 3. Physical Health | 42.85 | 9.10 | -.21** | -.09* |  |  |  |
|  |  |  | CI [-.28, -.13] | CI [-.17, -.01] |  |  |  |
|  |  |  |  |  |  |  |  |
| 4. Mental Health | 45.43 | 10.31 | -.37** | -.18** | -.06 |  |  |
|  |  |  | CI [-.44, -.30] | CI [-.25, -.10] | CI [-.14, .01] |  |  |
|  |  |  |  |  |  |  |  |
| 5. ERI Overcommitment | 2.62 | 0.60 | .46** | .13** | -.18** | -.45** |  |
|  |  |  | CI [.40, .52] | CI [.06, .21] | CI [-.25, -.10] | CI [-.51, -.39] |  |
|  |  |  |  |  |  |  |  |
| 6. Work-Family-Balance | 2.65 | 0.86 | .45** | .18** | -.22** | -.46** | .45** |
|  |  |  | CI [.38, .51] | CI [.11, .26] | CI [-.30, -.15] | CI [-.52, -.40] | CI [.39, .51] |
|  |  |  |  |  |  |  |  |
| CI=Confidence interval at the 95% level | | | | | | | |
| ^1^ negatively poled: higher values indicate lower quality | | | | | | | |
| Statistical significance: * p<0.1; ** p<0.05; *** p<0.01 | | | | | | | |

**S1: Correlation Matrix of subscales (N=231)**
